# Synthesis, Metal Ion Complexation and Computational Studies of Thio Oxocrown Ethers

**DOI:** 10.3390/molecules16108670

**Published:** 2011-10-14

**Authors:** Baki Çiçek, Ahmet Yıldız

**Affiliations:** Department of Chemistry, Faculty of Science and Literature, University of Balikesir, 10145, Balıkesir, Turkey

**Keywords:** thio-oxocrown ethers, conductometric, theoretical study, DFT, binary systems

## Abstract

The synthesis of some thio-oxocrown ether ligands, **B1** (1,4-dithio-12-crown-4), **B2** (1,7-dithio-12-crown-4), **B3** (1,7-dithio-15-Crown-5), **B4** (1,7-dithio-18-crown-6), **B5** (1,10-dithio-18-crown-6), **B6** (1,10-dithio-21-crown-7), under mild conditions, were reported. The ligands were characterized by FT-IR, ^1^H NMR and GC-MS spectroscopy. The formation of 1:1 ligand complexes with a variety of metal salts (Ag^+^, Ca^+2^, K^+^, Na^+^, Mg^+2^, Zn^+2^ and Fe^+2^) were investigated by a conductometric method in a 1:1 dioxane–water system at 25 °C, and the complexation constants (K_e_ = (Λ_MA*m*_ -Λ) / ((Λ-Λ_M*a*L*b*A*m*_) [L]) and free energy (∆G^o^= - RT lnK_e_) values are calculated. Details of the specific molecular interactions between the ligands and metals were proposed. We also performed DFT calculations to explain their geometrical properties, charges and frontier molecular orbitals.

## 1. Introduction

Synthetic macrocycles, in particular crown ethers, have been known for over three quarters of a century, although a real spate of publications in this area was observed in the late 1960s [1,2]. In that period, thousands of macrocyclic compounds were reported, and since then their number has increased markedly from year to year. Crown ethers contains “hard” ether–oxygen-bridges and show a binding preference toward “hard” metals (such as alkali and alkaline earth metal cation). The replacement of oxygen-bridge with “soft” sulfide or amine linkages can shift their preference towards “soft” heavy metal cations [3]. Thus, selectivity can be tuned by combining different hard/soft donor atoms in one ring system. In this regard oxo-thiocrown ethers are prepared and used as potential heavy-metal receptors. In this work, oxo-thiocrown ethers having different ring size or arrangement have been prepared and their complexation properties have been investigated with various metal cations by conductometry [4,5,6,7].

More recently, interest in differences in the properties of metal complexes caused by replacement of one or more thioether sulfurs with amine nitrogen atoms has been apparent in the literature [8,9]. In spite of this and the obvious connection between crown ethers and thiocrown ethers, until quite recently [10,11], less effort has been devoted to a comparison of O,S donor complexes with complexes of these other ligand systems. As part of our continuing interest in the properties of both acyclic and cyclic ligands that have thioether and other donors [12,13], we have examined the properties of some metal complexes of ligand L and the results are reported herein for the purpose of comparison with those from homothioether and mixed S,N donor macrocyclic ligand complexes [14].

Binary mixed aqueous solvents are frequently employed in broad areas of chemistry. Their applicability ranges from synthetic and mechanistic studies in organic chemistry to biophysical chemistry, with emphasis on molecular interactions in biologically significant structures. Stability constants of crown compound-action complexes are determined by various methods, such as potentiometry (with ion selective electrodes), polarography, voltammetry, spectrophotometry, NMR, calorimetry and solubility. The ability of macrocyclic polyethers (crown ethers) to form stable complexes with cations, mainly with alkali and alkaline earth cations, has spurred interest in these compounds [15,16,17]. Recently published studies include conductometric measurement of some electrolytes in non-aqueous solvents (tetrahydrofuran [18], tetrahydropyran [18], acetonitrile [19,20] and methanol [20]) in the presence of crown-ethers. Studies carried out in solutions of alkali metal salts and crown-ethers in acetonitrile and methanol showed decreases in conductivities of the solutions.

The object of the present work was to study the conductance behavior of AgNO_3_, CaCl_2_, KCl, NaCl, MgCI_2_, ZnCl_2_ and FeSO_4_ and with **B1** (1,4-dithio-12-crown-4), **B2** (1,7-dithio-12-crown-4), **B3** (1,7-dithio-15-Crown-5), **B4** (1,7-dithio-18-crown-6), **B5** (1,10-dithio-18-crown-6), **B6** (1,10-dithio-21-crown-7) in the dioxane/water mixtures (50%) at 25 °C. The study indicates 1:1 complex formation between the metal-ions and electrically neutral crown ethers. 

The experimental findings were also supported by theoretical computations. The gas phase molecular mechanical and quantum chemical calculations of **B1, B2, B3, B4, B5,** and **B6** were performed at B3LYP/6-31G(d) level with the help of the Gaussian03 program.

## 2. Results and Discussion

**Scheme 1:**
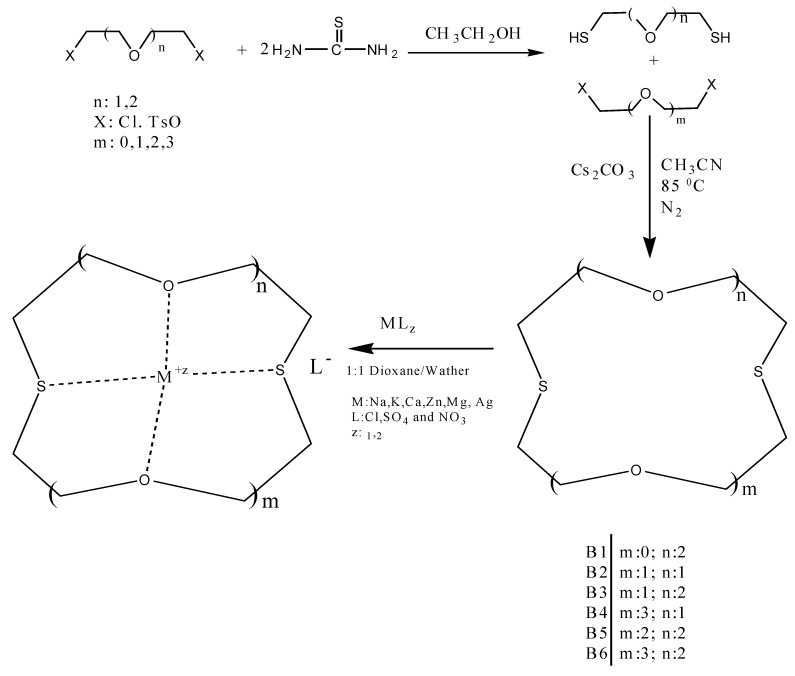
Syntheses of several oxo-thiocrown ethers (**B1**-**B6**) and complexation with metals.

In this work, several oxo-thiocrown ethers **B1**-**B6** with different ring sizes and structural isomers, were synthesized in acetonitrile under high dilution conditions by the S_N_2 reaction of di/tri-ethyleneglycol dithiols with mono/di/tri-ethyleneglycol dichlorides/ditosylates in the presence of CsCO_3_. From this the folowing oxo-thiocrown ether ligands were obtained in low (8%) to moderate (48%) to high yield (73%): **B1** (1,4-dithio-12-crown-4), **B2** (1,7-dithio-12-crown-4), **B3** (1,7-dithio-15-Crown-5), **B4** (1,7-dithio-18-crown-6), **B5** (1,10-dithio-18-crown-6), **B6** (1,10-dithio-21-crown-7). The oxo-thiocrown ethers (**B1**-**B5**) were previously reported by others; however **B6** is new to the best of our knowledge (Scheme 1). The reported procedure was modified slightly by replacing DMF with acetonitrile, which is easy to evaporate.

The oxo-thiocrown ethers (B1-B6) are hygroscopic materials, supported by the presence of O-H stretching vibrations at 3,500 cm^−1^. The dithiols used in the synthesis of oxo-thiocrown ethers were obtained by two different methods. Generally thio derivatives, such as dithiols or thio-crown ethers are expensive materials; hence ethyleneglycol derivatives are frequently used as cheap starting materials. In this regard, initially Method A was explored. This involves the reaction of di/tri-ethyleneglycol with thiourea in the presence of HCl, where dichlorides were generated *in situ*, followed by the addition of KOH. On the other hand Method B involves direct reaction of di/tri-ethyleneglycol dichlorides with thiourea in the presence of KOH. The both method gave moderate yields (30–36%). Dithiols were purified by using vacuum distillation and then characterized with FT-IR, ^1^H NMR and GC-MS. The S-H streching vibration for dithiols was characterized by the presence of band at 2,500 cm^−1^. 

For example, if we have prepared dithio-18-crown-6 using both ways, the product obtained from ditosylate is less than the product made from the dichloride because, the tosylate is a group that can be separated in S_N_2 mechanism under normal conditions but it is too hard to remove it from complex donor crown ethers. Especially, that’s hard to purify the molecules which have –S center oxygen, so their yields are lower.

Various methods to calculate stability constants from measurements of properties involving intrinsic factors, such as molar conductivity etc. have been described in the literature [21-23]. When an oxo-thiocrown ether ligand (L) forms a complex (M*_a_*L*_b_^m^*^+^) with a cation (M^m+^), the equilibrium equation can be written as:

*a* M*^m^*^+ ^+ *b*L ⇌ M*_a_*L*_b_^m^*^+^

αC_M _C_L_ -(1-α)C_M_ (1- α)C_M_ (1)

where M^m+^, L, and α are respectively the cation, ligand and fraction of free cations. Thus the equilibrium constants K_e_ of different ratios of complex formation were calculated using the following equations (2)–(13):

K_e_ = [M*_a_*L*_b_^m^*^+^] / [M*^m^*^+^]*^a^* [L]*^b^* (2)

C_M_ / C_L_ = 1 (3)

C_M_ = [M_a_*^m^*^+^] +[M*_a_*L*_b_^m^*^+^] (4)

C_L_ = [L_b_] +[M*_a_*L*_b_^m^*^+^] (5)

α = [M_a_^m+^] / C_M_ (6)

P = [M*_a_*L*_b_^m^*^+^] / C_M_ =K_e_[L_b_] / (1+ K_e_[L_b_] ) (7)

The observed conductivity, κ, is given by:

κ= κ_Ma_^m+^ +κ_M*a*L*b*_*^m+^* (8)

The molar conductivities are:

Λ_MA*m*_ = κ_MA*a*_*^m+^*_/_ [M_a_*^m^*^+^] (9)

Λ_M*a*L*b*_*^m+^* = κ_ M*a*L*b*_*^m+^* / [M*_a_*L*_b_^m^*^+^] (10)

Λ= κ / C_M_ (11)

Λ= αΛ_Ma_^m+^ + (1- α)Λ_M*a*L*b*_^m+^**(12)

As a result of Eq. (12), Eq. (2) can be transformed into:

K_e _= (Λ_Ma_^m+^ - Λ) / ((Λ-Λ_M*a*L*b*_*^m+^*) [L_b_] ) (13)

where [L_b_] = C_L_ - C_M_.P and [L_b_] = C_L_ - C_M_.(Λ_Ma_^m+^ - Λ)/(Λ_Ma_^m+^ - Λ_ M*a*L*b*_*^m+^*) and C_M_, C_L_ are the total concentrations of metal ion and crown ether, respectively; **[M_a_^m+^]**, **[L_b_]** and **[M*_a_*L*_b_^m^*^+^]**, are the concentrations of uncomplexed action, uncomplexed crown ether and complexed cation, respectively; P, is the experimental mole fraction of the complexed cation or the ligand, and a and b are the complexing degrees of both sides in the case of several degrees of complexing [eqns. (1)-(13)] **κ_Ma_^m+^**, **κ_M*a*L*b*_*^m+^***, are the observed conductivities of the electrolyte and the crown compound-electrolyte complex, respectively; **Λ_Ma_^m+^** and **Λ_M*a*L*b*_^m+^** are the designated molar conductivities of the electrolyte and the crown compound-electrolyte complex, respectively [21].

In the present work, considerable solvent effects of water were displayed for **B1** (1,4-dithio-12-crown-4), **B2** (1,7-dithio-12-crown-4), **B3** (1,7-dithio-15-crown-5), **B4** (1,7-dithio-18-crown-6), **B5** (1,10-dithio-18-crown-6), **B6** (1,10-dithio-21-crown-7), owing to differences in K_e_ depending on the solvent composition. The selectivity for a given salt is a factor not only of the crown ether, but of the solvent as well [21,24]. These various selectivities depend on the crown ether interactions with the cations governed by charge density. 

The complexation of the synthesized products with Na^+^ , K^+^ , Ca^2+^, Zn^2+^, Mg^2+^, Ag^+^ and Fe^2+^ was studied by conductometry in 50% dioxane-water at 25 °C and original results were obtained. The complexation selectivity of **B1** with Na^+^, K^+^, Ca^2+^, Zn^2+^, Mg^2+^, Ag^+^ and Fe^2+^ ions, were observed to be as follows: Na^+^ > Fe^2+^ > Mg^2+^ > Ca^2+^ > Ag^+^ > K^+^ > Zn^2+^. The best complexation of with **B1** with Na^+^ can be explained by the possibility of having sandwich molecules with more than one ion because its ring cavity is bigger.

The complexation sequence for **B2** was obtained as follows: Fe^2+^ > Ca^2+^ > K^+^ > Mg^2+^ > Na^+^ > Zn^2+^ > Ag^+^. The best complexation with **B2 **is shown by Fe^2+^ ion and the least is Ag^+ ^ion .

**B3** gave the sequence K^+^ > Ca^2+^ > Na^+^ > Zn^2+^ >Fe^2+^ > Ag^+^ > Mg^2+^. Thus, the highest association constant for complexation of **B3** was found for K^+^ ion. 

However, for **B4,** the order is Na^+^ > Zn^2+^ > Ca^2+ ^> Fe^2+^ > Ag^+^ > K^+^ > Mg^2+^. Hence, the best complexing with **B4** is Na^+^. 

Moreover, **B5,** gives the order K^+^ > Ag^+^ > Ca^2+^ > Zn^2+^ > Mg^2+^ > Na^+^ > Fe^2+^. The best complexing with **B5** is K**^+^**.

**B6,** gives the order Mg^2+ ^> Na^+ ^> Ca^2+ ^> K^+^ > Zn^2+ ^>Fe^2+^ > Ag^+^. The best complexing with **B6** is Mg^2+^.

All oxo-thio crown ethers **B1-B6** have systematic variations in the ring size of the crown ether ring from [12]crown-4 to [15]crown-5 to [18]crown-6 to [21]crown-7, respectively. Results of our alkali metal complexation study using oxo-thio crown ether derivatives are presented in Table 1. One of the most impressive results of this study is the dithio-12-crown-4 and dithio-18-crown-6 derivatives’ complexing ability in dioxane-water system. Some specific results were obtained by exchanging of oxygen and sulphur in the crown-ring. For example, for Ag^+ ^the best are **B1**, **B3**, **B2** and then, **B6** is lesser. Mg^++ ^has the highest complexing ability with **B6**, and it complexes with **B3 **and **B5.** This is caused by the conformational difference between the oxygens and sulphurs in the structure. The ligands which best complex with the metals were determined in this sequence: for NaCI and KCl **B1** is the best, for ZnCl_2_ **B3**, for MgCl_2_ **B6**, for FeSO_4_ and CaCl_2_ **B2**. 

### 2.1. Computational Results

The difficulty of the theoretical calculations can be attributed to the complication of supramolecular structures. Various quantum-chemical and force-field computations of crown ethers and of their metal complexes have been reported recently [24,26,27,28], even on the *ab initio* level [29,30] and the density functional level of theory (DFT) [31,32,33]. The structures of molecules play an especially significant role in determining their chemical properties. Therefore, we study the most stable geometries of the six crown ethers. In order to obtain the preferred conformers, the simulated anneling technique was employed initially [34]. The simulation protocol involving a heating time of 0.1 ps, followed by a 1 ps simulation at 3,000 K and cooling to 298 K within 50 ps was applied at AM1 self-consistent field molecular orbital level [35]. Quantum-mechanical calculations were carried out on initially geometry optimized structures at the DFT level using the Gaussian03 suite of programs [36]. The DFT methods are effective for the theoretical studies of supramolecular structures [37,38,39]. The B3LYP, a version of the DFT method, which uses Becke’s three-parameter functional (B3) [40] and includes a mixture of HF and DFT exchange terms associated with the gradient corrected correlation functional of Lee, Yang, and Parr (LYP) [41]. Hence, B3LYP method is used in this article to perform quantum chemical calculations.

**Table 1:** The Association Constants (K_e_) and Free Enthalpies (ΔG^θ^) of NaCI, KCI, CaCl_2_, ZnCl_2_, MgCl_2_, AgNO_3_ and FeSO_4 _with B1 (1,4-dithio-12-crown-4), B2 (1,7-dithio-12-crown-4), B3 (1,7-dithio-15-Crown-5), B4 (1,7-dithio-18-crown-6),B5 (1,10-dithio-18-crown-6) and B6 (1,10-dithio-21-crown-7), with (1:1) in %50 Dioxane/Water Mixtures at 25 °C.

Thiocrown ethers	Cation	K_e (1:1)_	LogK_e(1:1)_	-ΔG^θ^_1:1_
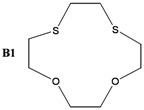	Na^+^	159005,00	5,201411	7091,71
	K^+^	4659,09	3,668301	5001,44
	Ca^2+^	21002,62	4,322273	5893,08
	Zn^2+^	4134,76	3,61645	4930,72
	Mg^2+^	22862,18	4,359118	5943,31
	Ag^+^	15602,02	4,193181	5717,07
	Fe^2+^	63856,70	4,805206	6551,52
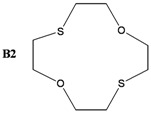	Na^+^	24556,00	4,390158	5985,63
	K^+^	1177370,00	6,070913	8277,21
	Ca^2+^	1334785,00	6,125411	8351,51
	Zn^2+^	9608,34	3,982648	5430,02
	Mg^2+^	113650,30	5,055571	6892,87
	Ag^+^	6486,80	3,812031	5197,40
	Fe^2+^	2542753,00	6,405304	8733,12
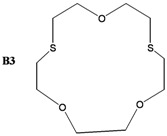	Na^+^	128719,00	5,109643	6966,59
	K^+^	1073250,00	6,030701	8222,38
	Ca^2+^	516059,00	5,712699	7788,81
	Zn^2+^	66872,94	4,82525	6578,85
	Mg^2+^	-	-	-
	Ag^+^	15602,07	4,193182	5717,07
	Fe^2+^	30157,18	4,479391	6107,29
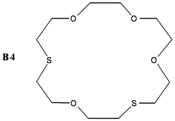	Na^+^	136555,00	5,135308	7001,58
	K^+^	217,76	2,337978	3187,65
	Ca^2+^	24444,83	4,388187	5982,94
	Zn^2+^	58836,12	4,769644	6503,03
	Mg^2+^	-	-	-
	Ag^+^	246,23	2,391341	3260,40
	Fe^2+^	3701,47	3,568374	4865,20
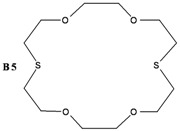	Na^+^	4144,79	3,617503	4932,18
	K^+^	57052,90	4,756278	6484,81
	Ca^2+^	37945,99	4,579166	6243,33
	Zn^2+^	31015,46	4,491578	6123,91
	Mg^2+^	22825,90	4,358428	5942,37
	Ag^+^	54208,9	4,734071	6454,529
	Fe^2+^	390.0	2,591065	3532,71
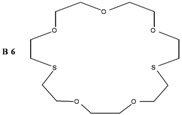	Na^+^	127313,00	5,104873	6960,09
	K^+^	22056,90	4,343544	5922,08
	Ca^2+^	37945,99	4,579166	6243,33
	Zn^2+^	11118,95	4,046064	5516,49
	Mg^2+^	517167,30	5,713631	7790,08
	Ag^+^	298,72	2,475264	3374,83
	Fe^2+^	6359,96	3,803454	5185,71

Corr.Coeff: Na^+^ :0,9993, K^+^: 0,9995, Ca^2+^: 0,9997, Zn^2+, ^: 0,9995, Mg^2+^:0,9994, Ag^+^: 0,9996 and Fe^2+^:0,9997

The optimized structures for the crown ethers studied herein in their gorund states were obtained and are shown in Table 2. The dihedral angles were also calculated to analyze the planarity of molecules. The ring-contracted crown ether **B5** has the biggest dihedral angle, Ø_SOOS_ = 42.51° and indicates the most distorted structures for series of compounds. The results indicate that when a methylene between two adjacent oxygen atoms of a crown ether is reduced, the rigidity of the crown ring is increased and the structure of the crown ether shows a drastic change in the atoms’ disorder. However, compared to the conventional crown ethers **B2** and **B3**, the ring-contracted crown ethers all depict a relatively good oxygen planarity because the crown ether rings became more soft and relaxed for the changing of the methylene-chain length between sulfur and oxygen atoms. The calculated real vibrational frequencies of **B1, B2, B3, B4, B5,** and **B6** at B3LYP/6-31G(d) level show that the molecules are located as a minimum on potential energy surfaces.

The highest occupied molecular orbital (HOMO) and lowest unoccupied molecular orbital (LUMO) of molecules are quite important to define its reactivity. Fukui *et al*. were the first to recognize this. E_HOMO_ is often called with the electron donating ability of the molecules [42]. E_HOMO_ depicts that the molecular ability in donating electrons to appropriate acceptor molecules with low energy, empty molecular orbital. On the contrary. E_LUMO_ indicates the ability of the molecule to accept electrons. The lower value of E_LUMO_, indicates that the molecule would accept electrons. Hence, concerning the value of the energy gap, ΔE(E_LUMO_–E_HOMO_), higher values of ΔE will provide lower reactivity to a molecule. Lower values of ΔE will indicate the higher reactivity of the molecules, because the energy to remove an electron from the HOMO to the LOMO orbital will be low [43,44,45,46]. Frontier molecular orbital diagrams and energies of **B1, B2, B3, B4, B5,** and **B6**, such as E_LUMO_, E_HOMO_, and ΔE (in eV) estimated by the B3LYP/6-31G(d) levels are represented in Table 2. 

**Table 2:** Frontier molecular Orbital Diagrams and Energies of I and II by the B3LYP/6-31G(d) (isovalue: 0.02).

	HOMO	LUMO	*ΔE*
**B1**	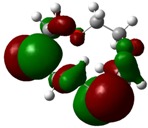	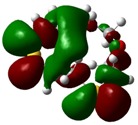	
E (*eV*)	−0.219	0.030	0.249
**B2**	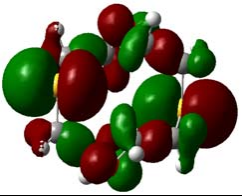	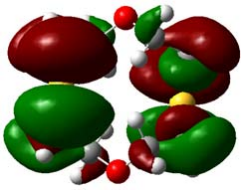	
E (*eV*)	−0.209	0.042	0.251
**B3**	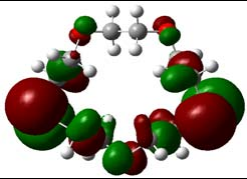	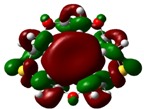	
E (*eV*)	−0.228	0.024	0.249
**B4**	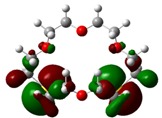	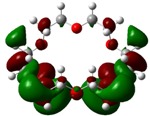	
E (*eV*)	−0.202	0.053	0.255
**B5**	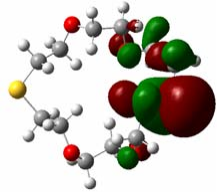	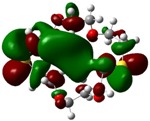	
E (*eV*)	−0.217	0.031	0.248
**B6**	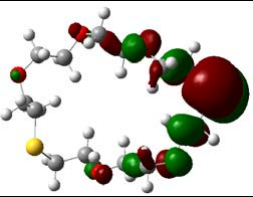	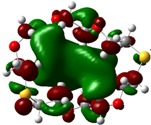	
E (*eV*)	−0.229	0.019	0.248

The values of E_HOMO_ show the ranking: B4 > B2 > B5 > B1 > B3 > B6 for this property. In addition, the values of ΔE show this order: B4 > B2 > B1 ≥ B3 > B5 ≥ B6. As can be seen from the HOMO and LUMO pictures in Table 2, the majority of HOMO and LUMO are found on the donor atoms in the crown ether ring, suggesting the high electron-donating ability of heteroatoms.

Table 3 shows the atomic charges from natural population analyses (NPA) for **B1, B2, B3, B4, B5,** and **B6**. The calculated charges on the sulfur atoms are considerable more positive than that on oxygen atoms. Results primarily indicate the role of delocalized oxygen charges on the torsional barrier of gauche oxyethylene units of crown ethers which could optimize charge densities and therefore the preferred conformations. This also influenced the polarization balances of the rest of atoms in a macromolecule governing the power of macrocycle-cation interactions.

**Table 3:** NBO Charges on Oxygen and Sulfur Atoms of B1, B2, B3, B4, B5, and B6.

Complex	Charges on Oxygen atoms	Charges on Sulfur atoms
**B1**	−0.591 / −0.599	0.196 / 0.199
**B2**	−0.591 / −0.591	0.186 / 0.186
**B3**	−0.578 / −0.579 / −0.579	0.207 / 0.207
**B4**	−0.573 / −0.573 / −0.576 / −0.583	0.227 / 0.227
**B5**	−0.580 / −0.581 / −0.584 / −0.600	0.201 / 0.211
**B6**	−0.575 / −0.580 / −0.580 / −0.582 / −0.582	0.210 / 0.214

## 3. Experimental

### 3.1. General

The starting materials triethyleneglycoldiol, diethyleneglycoldiol, triethyleneglycol dichloride, diethyleneglycol dichloride, triethyleneglycol ditosylate diethyleneglycol ditosylate, ethylene ditosylate, Cs_2_CO_3_ and thiourea were purchased from Aldrich or Merck. IR spectra were recorded on a Perkin Elmer BX 2 FTIR spectrophotometer using KBr pellets. ^1^H-NMR spectra were recorded on a Varian 400 MHz spectrometer in CDCl_3_ and chemical shifts are reported relative to Me_4_Si used as an internal standard. Mass spectra were obtained using a Shimadzu GS-MS-QP2010 spectrometer. Melting points were measured on an Elektotermal 9200 apparatus.

#### 3.1.1. The Synthesis of Dithiols

*Triethyleneglycoldithiol *from triethylene glycol (Method A): triethyleneglycol (24 mL, 0.18 mol) and thiourea (30 g, 0.39 mol) were heated in HCl (150 mL) for 40 hours under reflux. Then, the reaction mixture was left to cool to room temperature. A 3.3 M aqueous KOH (300 mL) was added carefully to the reaction mixture. The solution was left to cool to room temperature, and then organic products were extracted into ether (2 × 150 mL). Combined extracts were dried over MgSO_4_, the solids were filtered and the solvent was evaporated under reduced pressure to give a crude material as liquid. Upon distillation (5 mm Hg, 140 °C) the title product (12.02 g, 36%) was obtained as a yellow liquid. ^1^H-NMR (δ, CDCl_3_): 1.55 ppm (2H, t, SH), 2.73 ppm (4H, t, -CH_2_SH), δ 3.65 ppm (4H, s, ‑OCH_2_CH_2_O-) 3.75 ppm (4H, t, OCH_2_CH_2_SH); FT-IR (ν cm^−1^, KBr): 2553 (S-H), 1113 (C-O-C). 

From trietyleneglycol dicloride: (Method B): triethyleneglycol dichloride (25 mL, 0.15 mol) and thiourea (30 g, 0.39 mol) were heated in ethanol (150 mL) for 50 hours under reflux. Then, the reaction was left to cool to room temperature. A 1.75 M aqueous KOH (200 mL) was added carefully to the reaction mixture. The solution was left to cool to room temperature; organic products were extracted into ether (2 × 100 mL). Combined extracts were dried over MgSO_4 _the solids were filtered off and the solvent was evaporated under reduced pressure to give a crude material as liquid. Upon distillation (5 mmHg, 140 °C) the title product (8.92 g, 30%) was obtained as yellow liquid. FT-IR (ν cm^−1^, KBr): 2555 (S-H), 1115 (C-O-C). 

*Dietyleneglycoledithiols* (by Method B). Starting with diethyleneglycol dichloride (20 mL, 0.17 mol), thiourea (30 g, 0.39 mol) and KOH (20 gr, 0.35 mol) the title product (9.03 g, 36%) was obtained as a clear liquid upon distillation (4 mm-Hg, 143 °C). ^1^H-NMR (δ, CDCl_3_): 1.55 (2H, t, SH), 2.65 (4H, t, -CH_2_SH), 3.55 (4H, t, -OCH_2_CH_2_SH). FT-IR (ν cm^−1^, KBr): 2557 (S-H), 1110 (C-O-C).

#### 3.1.2. The Synthesis of Oxo-thiocrown Ethers

General procedure: ditosylate/dichloride was added to a suspension of dithiol (1.1 equiv.) and Cs_2_CO_3_ (5.0 equiv.) in acetonitrile (250 mL). The mixture was then heated under reflux for 24 h under a nitrogen atmosphere. The reaction was left to cool to room temperature and then the precipitates were filtered. The solvent was evaporated under reduced pressure to give a crude material as solid. Distilled water (100 mL) was added and organics were extracted into benzene-chloroform (10:1 v/v). Combined extracts were dried over MgSO4 and then evaporated under reduced pressure. Purification by column chromatography (benzene/ethyl acetate; 1:10 v/v) afforded the title compounds.

*1,4-Dithio-7,10-dioxocyclododecane* (**B1**): Starting with ethylene glycol ditosylate (1.57 g, 4.24 mmol), triethyleneglycol dithiol (0.84 g, 4.66 mmol) and Cs_2_CO_3_ (7 g, 21.2 mmol) the title compound (0,65 g, 73%) was obtained as thick oil. ^1^H-NMR (δ, CDCl_3_): 2.78 (8H, t, -SCH_2_-CH_2_O-), 3.00 (4H, s, ‑SCH_2_CH_2_S-), 3.60 (4H, s, -OCH_2_CH_2_O-), 3.78 (4H, t, -OCH_2_CH_2_S-); FT-IR (ν cm^−1^, KBr): 1177 (C-O-C), 684 (C-S-C). GC-MS: (*m/z*) M^+^: 208.34 (100%).

*1,7-Dithio-4,10-dioxocycloododecane* (**B2**): Starting with diethylene glycol dichlorides (2.34 mL, 0.02 mol), diethyleneglycol dithiol (2.76 g, 0.02 mol) and Cs_2_CO_3_ (35 g, 0.105 mol) the title compound (1.97 g, 45%) was obtained as a thick oil. ^1^H NMR (δ, CDCl_3_): 2.90 (8H, t, -SCH_2_-CH_2_O-), 3.79 (4H, t, -OCH_2_CH_2_S-). FT-IR (ν cm^−1^, KBr): 1111 (C-O-C), 661 (C-S-C). GC-MS: (*m/z*) M^+^: 208.34 (100%).

*1,7-Dithio-4,10,13-trioxocyclopentadecane* (**B3**): Starting with diethylene glycol ditosylate (1.75 gr, 4.24 mmol), triethyleneglycoldithiol (1.26 g, 6.99 mmol) and Cs_2_CO_3_ (7 g, 21.2 mmol) the title compound (0,88 g, 48%) was obtained as yellow crystal. Melting point: 63–64 °C. ^1^H-NMR (δ, CDCl_3_): 2.95 (8H, t, -SCH_2_-), 3.65 (4H, s, -OCH_2_CH_2_O-), 3.75 (8H, t, -OCH_2_CH_2_S-). FT-IR (ν cm^−1^, KBr): 1176 (C-O-C), 665 (C-S-C). GC-MS: (*m/z*) M^+^: 252.40 (100%).

*1,7-Dithio-4,10,13,16-tetraoxocyclooctadecane* (**B4**): Starting with tetraethyleneglycol dichloride (1.95 mL, 0.01 mol), diethyleneglycol dithiol (1.38 g, 0.01 mol) and Cs_2_CO_3_ (17.1 g, 0.05 mol) the title compound (0,24 g, 8%) was obtained as a thick oil. ^1^H-NMR (δ, CDCl_3_): 2.80 (8H, t, -SCH_2_-), 3.65 (8H, t, -OCH_2_CH_2_O-), 3.75 (8H, t, -OCH_2_CH_2_S-). FT-IR (ν cm^−1^, KBr): 1115 (C-O-C), 665 (C-S-C) GC-MS: (*m/z*) M^+^: 208.34 (100%).

*1,10-Dithio-4,7,13,16-tetraoxocyclooctadecane* (**B5**): Starting with triethyleneglycol ditosylate (2.91 g, 6.36 mmol), triethyleneglycol dithiol (1.26 g, 6.99 mmol) and Cs_2_CO_3_ (10.30 g, 31.8 mmol) the title compound (0.27 g, 14 %) was obtained as a yellowish solid. When triethyleneglycol dichloride was used, the yield was improved to 38%. Melting point: 93–94 °C. ^1^H-NMR (δ, CDCl_3_): 2.80 (8H, t, ‑SCH_2_-), 3.60 (8H, t, -OCH_2_CH_2_O-), 3.75 (8H, t, -OCH_2_CH_2_S-). FT-IR (ν cm^−1^, KBr): 1176, 1114 (C-O-C), 663 (C-S-C). GC-MS: (*m/z*) M^+^: 296.45 (100%).

*1,10-Dithio-4,7,13,16-pentaoxocyclooctadecane* (**B6**): Starting with tetraethylene glycol dichloride (1.95 mL, 0.01 mol), triethyleneglycol dithiol (1,82 g, 0.01 mol) and Cs_2_CO_3_ (17.1 gr, 0.05 mol) the title compound (0.66 g, 19%) was obtained as a thick oil. ^1^H-NMR (δ, CDCl_3_): 2.75 ppm (8H, t, ‑SCH_2_-CH_2_O-), 3.54 ppm (12H, p, -OCH_2_CH_2_O-), 3.69 ppm (48, t, -OCH_2_CH_2_S-). FT-IR (ν, cm^−1^, KBr): 1119 (C-O-C), 665 (C-S-C). GC-MS: (*m/z*) M^+^: 340.50 (100%).

### 3.2. Conductimetric Method

Merck grade alkali halides (NaCl and KCl) were recrystallized three times from a conductivimetric grade water-ethanol mixture and Merck grade AgNO_3_, CaCl_2_, MgCI_2_, ZnCl_2_ and FeSO_4_) were used directly. All of the salts were heated below their decomposition temperature at reduced pressure to remove traces of water in the crystal structure. Bi-distilled water was redistilled from alkaline permanganate. All solutions were prepared in dry glassware and transferred into the pre-dried conductivity cell. 

All conductances were measured at 25 ± 0.05 °C. The measuring equipment consisted of a glass vessel (Ingold type) with an external jacket connected to a thermostatted water-bath (25 ± 0.05 °C) and a conductivity cell (Cole Parmer 19050-66) with a conductometer (Suntex Model SC-170).

The cell constant was determined to be 0.769 cm^−1^ at 25 ± 0.05 °C by measuring the conductivity of aqueous KCl solutions of different concentrations [21,25]. A value of the molar conductivity of the pure metal ion solution (**Λ_MA_**) was obtained at the appropriate electrolyte concentration before adding any ligand.

## 4. Conclusions

In conclusion, various macrocyclic hosts with diverse affinity towards the metal ions and other funtions have been readily synthesized by changing the combinations of the oxygen and sulfur atoms forming the ring framework. The synthesized thiocrown ethers exhibited remarkably high binding constants in metal ion transport experiments [21,24,26]. 

The synthesized macrocyclic ethers exhibit different binding ability orders towards the examined metal ions. Although the effect of various synthesized thiocrown ethers on the transport ability of the studied metal cations was not investigated systematically, preliminary results show that thiocrown ethers may possess an advantage over crown ethers containing only oxygen atoms in the cyclic framework for metal binding, because the introduction of a sulfur atom into a crown ring gives rise to a favorable entropic change upon complexation with the metal ions. The results strongly suggested that the binding results from the synergistic coordination with polarization balance of the oxygen and sulfur atoms to the metal ions. 

As a result, we have demonstrated that a complementary theoretical and experimental approach can provide important information on oxo-thiacrown ethers complexation with various metal ions (Ag^+^, Ca^+2^, K^+^, Na^+^, Mg^+2^, Zn^+2^ and Fe^+2^), essential elements in a wide variety of processes in biological systems. The position of sulfur atom in the crown ether ring plays an important role in determining the ethers’ properties, as it can not only change the cavity size, but may also cause a disorder of the atom arrangement. Less-symmetrical crown ethers show remarkable recognition and binding properties towards specific metal cations compared to the highly-symmetrical crown ethers (Table 1, Table 2, and Table 3). It is believed that both of them will be widely applied in the future.
